# A Selenone-Functionalized Polyhedral Oligomeric Silsesquioxane for Selective Detection and Adsorption of Hg^2+^ ions in Aqueous Solutions

**DOI:** 10.3390/polym11122084

**Published:** 2019-12-13

**Authors:** Hailong Liu, Zixu Chen, Shengyu Feng, Dengxu Wang, Hongzhi Liu

**Affiliations:** 1 National Engineering Technology Research Center for Colloidal Materials & Key Laboratory of Special Functional Aggregated Materials, Ministry of Education, School of Chemistry and Chemical Engineering, Shandong University, Jinan 250100, China; 2 Shandong Dongyue Organosilicon Materials Co. Ltd., Zibo 256401, China

**Keywords:** polyhedral oligomeric silsesquioxane, selenone, sensors, detection of Hg^2+^, adsorption of Hg^2+^, water treatment

## Abstract

Developing novel functional polyhedral oligomeric silsesquioxane (POSS) for various applications is highly desirable. Herein we present the first example of a novel selenone-functionalized POSS (POSS-Se) by treating an imidazolium-containing POSS with selenium powder under mild condition. The structure of POSS-Se was characterized by FT-IR, ^1^H NMR, ^13^C NMR, ^29^Si NMR, and elemental analysis. Acid treatment of POSS-Se results in a hydrophilic red-orange colored solid, which is highly sensitive and selective for the detection of Hg^2+^ ions in aqueous solutions by visually observing the color change to pale yellow, and to white. Interestingly, POSS-Se has no activity on this detection. This finding is due to the Se–Se formation by acid-treatment and subsequent coordination-induced cleavage upon the addition of Hg^2+^ ions. The detection behavior can be precisely monitored by a “turn-on” fluorescence phenomenon with the limit of detection (LOD) of 8.48 ppb, comparable to or higher than many reported Hg^2+^ sensors. Moreover, POSS-Se demonstrates a selective and efficient adsorption of Hg^2+^ ions with a maximum capacity of 952 mg g^–1^. The value is higher than most reported adsorbents for Hg^2+^ ions, typically thiol and/or thioether functional materials, indicating its promise as an efficient adsorbent for the selective removal of Hg^2+^ ions from industrial wastewater. This work may open up new horizons for the exploration of selenium-containing functional POSS.

## 1. Introduction

The field of silsesquioxane chemistry, especially cage-like polyhedral oligomeric silsesquioxanes (POSSs), has developed dramatically owing to their remarkable properties and extensive applications in recent years [1,2,3,4,5,6,7]. Compared to conventional organic molecules and polymers, POSS and their derivatives (typically T_8_, R_8_Si_8_O_12_) possess many unique characteristics, such as rigid, high symmetric, well-defined, and ideal inorganic–organic hybrid structures, nanoscopic size (1–3 nm) and high functionality, etc, and thus having intriguing advantages, such as high thermal stability, mechanical properties (e.g., strength, modulus, rigidity), and improved quantum yields [1,2,3,4,5,6,7,8]. By virtue of these merits, they have been widely applied in many fields, including drug delivery [9], wastewater treatment [10,11], chemical sensors [12,13,14,15,16], energy transfer [17], bioimaging [18], chromatography [19], polymer electrolytes [20], catalysis [21,22,23], and membranes [24], etc. To realize these applications, it is crucial to incorporate various functionality into the POSS derivatives and/or materials. For example, Ervithayasuporn et al. decorated anthracene groups on the peripheral surface of the octameric silsesquoxane cage, making the material exhibit anion identification and distinguish up to four ions (F^–^, OH^–^, CN^–^, PO_4_^3–^) [12]. Hong et al. attached bis(diphenylphosphino)amine ligand to POSS, affording a solubility-enhancing material for catalytic ethylene trimerization and tetramerization [23]. By combining imidazolium with POSS units, the POSS-based ionic liquids can be utilized to detect nitroaromatic explosives [15] and as solid polymer electrolytes [20]. Although many POSS-based functional materials are available, the variety is still small compared to the vast carbon-based functional materials so it is still highly desirable to develop more POSS-based materials with novel structures and unique properties.

Selenium, a member of group XVI of the periodic table, is an essential element in the human body with potential antioxidant properties [25]. Selenium-containing compounds and polymers have found applications in many areas, including organic synthesis, biochemistry, semiconductors, and ligand chemistry [26,27,28]. For example, selenium-containing polymers can potentially serve as mild-responsive drug delivery vehicles and artificial enzymes [25]. Han et al. developed a near-IR reversible fluorescent probe modulated by selenium for monitoring peroxynitrite and imaging in living cells [29]. Zhao et al. used a bifunctional selenide as the catalyst for the synthesis of trifluoromethylthiolated tetrahydronaphthalenes [30]. Son et al. presented submicro-polymer particles bearing imidazoline-2-selenone for the selective color-sensing of halogens and mercury ions [31]. In particular, organoselenium compounds as fluorescent probes for various analytes (e.g., thiols, biomolecules, mercury ions) possess certain advantages over N-, O-, and S-based fluorescent probes due to the unique properties of selenium [32]. Although organoselenium chemistry has become a well-established field of research, there is still no report on the exploration of selenium-containing functional POSS materials.

**Scheme 1:**
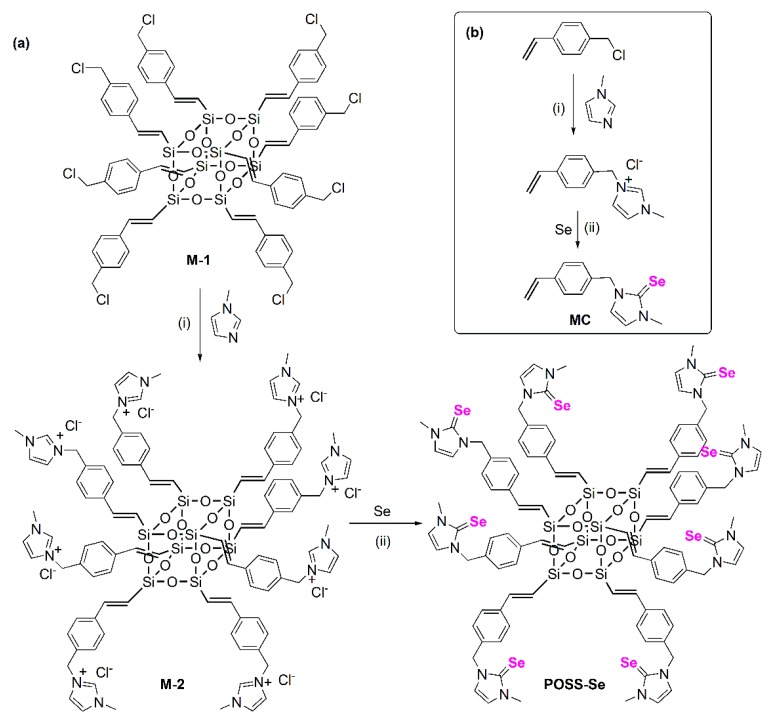
Synthetic routes of octa((4-benzyl imidazoline-2-selenone)ethenyl)silsesquioxane (POSS-Se) (**a**) and the model compound (MC) (**b**). (i) chloroform, reflux, 24 h; (ii) K_2_CO_3_, room temperature, 48 h (6 h for MC).

Herein, we combine the selenone groups with POSS units and present the first example of a selenone-functionalized POSS (POSS-Se) by the direct reaction of an imidazolium-functionalized POSS with selenium powder (Scheme 1). The resultant compound, POSS-Se, exhibits a high sensitivity and selectivity towards mercury ions (Hg^2+^) among various other metal ions after acid treatment. The detection process can not only be visualized by observing the color change of acidic POSS-Se, but also be precisely monitored by a “turn-on” fluorescence phenomenon. Moreover, the adsorption behavior of POSS-Se towards Hg^2+^ ions was investigated and it shows a high adsorption capacity, indicating that it can be utilized as a promising candidate for the removal of Hg^2+^ ions from aqueous solutions.

## 2. Materials and Methods

### 2.1. Materials and Characterization

Unless otherwise noted, all reagents were obtained from commercial suppliers and used without further purification. Octa((4-benzylchloride)ethenyl)silsesquioxane (M-1) was synthesized according to the method described in the previous report [33]. Fourier transform infrared (FT-IR) spectra were measured via KBr pellet technique within a 4000 to 400 cm^−1^ region on a Bruker TENSOR-27 infrared spectrophotometer (Ettlingen, Germany). ^1^H NMR, ^13^C NMR, and ^29^Si NMR spectra were measured on a Bruker AVANCE-400 NMR spectrometer (Rheinstetten, Germany) using CDCl_3_ or *d*_6_-DMSO as the solvent and without tetramethylsilane (TMS) as an internal reference. Elemental analyses were conducted using an Elementar vario EL III elemental analyzer (Munich, Germany). Thermogravimetric analysis (TGA) was performed under N_2_ using a TA SDTQ600 (New Castle, DE, USA) at a temperature range of room temperature to 600 °C with a heating rate of 10 °C min^−1^. The concentration of Hg^2+^ ions was measured by a GBC 923B-model atomic absorption spectrophotometer (Melbourne, Australian) or inductively coupled plasma mass spectrometry (ICP-MS) (Shelton, CT, USA). The fluorescent spectra of the samples were determined with a Hitachi F-7000 fluorescence spectrophotometer (Tokyo, Japan) using a monochromated Xe lamp as an excitation source. 

### 2.2. Synthesis of Octa((4-benzyl imidazolium chloride)ethenyl)silsesquioxane (M-2)

In a three-necked bottle, M-1 (1.632 g, 1 mmol) and 1-methylimidazole (1.313 g, 16 mmol) were dissolved in chloroform (15 mL). After reflux for 24 h, the resulting solution was allowed to cool to room temperature. The precipitate was filtered, washed with chloroform, collected, and dried under vacuum at 70 °C for 6 h. The product M-2 was afforded as a white solid (2.19 g, yield: 96%). IR (KBr pellet cm^−1^): 3136, 3057, 1606, 1567, 1450, 1419, 1383,1361, 1332, 1295, 1201, 1102, 1017, 883, 741, 659, 614, 548, 470. ^1^H NMR (400 MHz, DMSO) δ 9.67 (s, 8H), 7.94 (s, 8H), 7.82 (s, 8H), 7.64 (d, 16H), 7.51 (d, 16H), 7.37 (d, 8H), 6.54 (d, 8H), 5.54 (s, 8H), 3.89 (s, 24H). ^13^C NMR (100 MHz, DMSO): δ 149.1, 137.3, 136.6, 129.3, 128.0, 124.4, 122.8, 118.2, 51.7, 36.4. ^29^Si NMR (75 MHz, DMSO): δ −73.13.

### 2.3. Synthesis of Octa((4-benzyl imidazoline-2-selenone)ethenyl)silsesquioxane (POSS-Se)

In a three-necked bottle, M-2 (1.143 g, 0.5 mmol) was dissolved in methanol (30 mL). Potassium carbonate (3.317 g, 24 mmol) and selenium powder (0.947 g, 12 mmol) were added and the reaction mixture was stirred at room temperature for 48 h. The crude product was obtained after filtration and evaporating the solvent. Then 50 mL of water was poured into the crude product and the white solid was precipitated from the aqueous phase. The precipitation was filtered and washed with water. The product POSS-Se was dried under vacuum at 70°C for 6 h and afforded as a white solid (1.25 g, yield: 95%). IR (KBr pellet cm^−1^): 3125, 3089, 2933, 1678, 1606, 1566, 1510, 1460, 1395, 1357, 1234, 1199, 1122, 830, 760, 712, 668, 564, 453. ^1^H NMR (400 MHz, CDCl_3_) δ 7.34 (s, 8H), 7.27 (s, 8H), 7.20 (d, 16H), 6.91 (d, 16H), 6.78 (d, 8H), 6.25 (d, 8H), 5.35 (s, 8H), 3.72 (s, 24H). ^13^C NMR (100 MHz, CDCl_3_): δ 156.6, 137.1, 136.2, 128.5, 127.2, 120.3, 118.5, 111.8, 109.7, 52.9, 37.4. ^29^Si NMR (75 MHz, CDCl_3_): δ −79.48. Anal. Calcd for C_104_H_104_N_4_Se_8_O_12_Si_8_: C, 47.56; H, 3.99; N, 8.53. Found: C, 46.79; H, 4.09; N, 8.46. 

### 2.4. Detection of Hg^2+^ Ions by POSS-Se in Aqueous Solutions

Before the detection of Hg^2+^, POSS-Se was acidified by hydrochloric acid solution. POSS-Se (1 g) was added to 1.0 M HCl (40 mL) and held for 12 h at room temperature. The precipitate was retrieved by centrifugation, washed by water and dried under vacuum at 70 °C for 6 h. POSS-Se (HCl) was afforded as a red-orange solid.

The typical procedure for the detection of Hg^2+^ by POSS-Se (HCl): Dried POSS-Se (HCl) (2.5 mg) was added into deionized water (25 mL) and the resulting suspension (0.1 mg/mL) was dispersed with an ultrasonic dispersion instrument. To explore the sensing properties of POSS-Se (HCl) towards Hg^2+^, the fluorescence spectra of the resulting suspensions were recorded by the fluorescence analyzer upon the addition of successive concentrations of HgCl_2_ (0–100 ppm) in aqueous solutions.

Selective detection of Hg^2+^: The fluorescence emission spectra of POSS-Se (HCl) suspension (0.1 mg/mL) were first measured upon the addition of 100 ppm of metal ions with various cations, including Zn^2+^, Cr^3+^, Ca^2+^, Mn^2+^, Pb^2+^, and Cu^2+^. Then the competitive experiment was conducted by recording the fluorescent spectra of the POSS-Se (HCl) suspensions (0.1 mg/mL) in the coexistence of equivalent Hg^2+^ and other interfering metal ions (100 ppm).

### 2.5. Calculating the Limit of Detection (LOD) 

The limit of detection (LOD) was calculated based on the fluorescence titration. The standard deviation of a blank measurement was achieved by measuring the fluorescence emission spectrum of POSS-Se (HCl) suspension in water 6 times. To obtain the slope, the ratio of emission intensity at 385 nm was plotted as a concentration of Hg^2+^ ions. The LOD was calculated using the following Equation (1).

LOD = 3 × σ/K(1)

σ = Standard deviation of blank measurement; K = Slope between the ratio of emission intensity versus the concentration of Hg^2+^ ions.

### 2.6. Adsorption of Hg^2+^ Ions by POSS-Se in Aqueous Solutions

All the adsorption experiments of Hg^2+^ ions were conducted by POSS-Se suspension in water (1 mg mL^−1^) at room temperature. After the adsorption, the residual concentration of Hg^2+^ ions was measured by the atomic absorption spectrophotometer or ICP-MS. 

The effect of initial Hg^2+^ concentration was investigated at a series of concentrations including 100, 200, 500, 700, 1000, 2000, 3000, 4000, and 5000 ppm for 24 h equilibrium time in the presence of POSS-Se (1 mg mL^−1^).

The effect of contact time was investigated by an aqueous solution of Hg^2+^ ions (1000 ppm) and POSS-Se (1 mg mL^−1^) and the concentration of Hg^2+^ ions were stirred at 1, 2, 3, 4, 5, 12, 24, 36, and 48 h.

The removal efficiency of Hg^2+^ ions was estimated by using POSS-Se (1 mg mL^−1^) to remove Hg^2+^ ions with a concentration in the range of 50–5000 ppm in aqueous solutions. The effect of the co-existence of various metal ions, including Zn^2+^, Cr^3+^, Ca^2+^, Mn^2+^, Pb^2+^, and Cu^2+^ on the removal efficiency of Hg^2+^ was further measured by using POSS-Se (1 mg mL^−1^) to remove Hg^2+^ ions (100 ppm) in the presence of the competitive metal ions with a concentration of 100 ppm in aqueous solutions.

### 2.7. Adsorption Equilibrium and Kinetics

The adsorption equilibrium isotherm and relevant parameters were used to determine the adsorption mechanism and modeled using the Langmuir models (Equation (2)).

(2) $$\frac{C_{e}}{Q_{e}}\;=\;\frac{1}{Q_{m}}\;\cdot {\;K}_{L}\;+\;\frac{1}{Q_{m}}\cdot C_{e}$$

where *K_L_* (L mg^–1^) and *Q_m_* (mg g^−1^) represent the Langmuir constant and the maximum adsorption capacity as calculated by the Langmuir model.

The adsorption kinetics was explored using the pseudo-second-order adsorption models. The linear forms of these models are expressed according to Equation (3).

(3) $$\frac{t}{Q_{t}}\;=\;\frac{1}{k_{2}Q_{e}^{2}}\;+\;\frac{t}{Q_{e}}$$

where *Q_e_* and *Q_t_* (mg g^−1^) are the saturated uptake and the uptake at time t (h), respectively; k_2_ is the adsorption rate constant of the pseudo-second-order-model.

### 2.8. Recyclability of POSS-Se

The recyclability of POSS-Se towards Hg^2+^ ions was evaluated by considering the desorption and re-adsorption capability. The first adsorption experiment was performed with 5 mL of suspension in water containing Hg^2+^ ions (1000 ppm) and POSS-Se (1 mg mL^−1^) for 3 h. Then the suspension was centrifuged. After removing the supernatant, 5 mL of deionized water was added. The resultant suspension was dispersed with ultrasonic dispersion instrument and centrifuged again. The washing and centrifugation process were repeated three times, affording the adsorbed product, POSS-Se+ Hg^2+^. Then the desorption experiment was performed by adding 12 mL of sodium diethyldithiocarbamate (DDTC-Na) in aqueous solution (90 mg mL^−1^) and 21 mL of CH_3_CN to the adsorbed product and subsequently dispersing under ultrasound for 30 min. After centrifuging the resulting suspension and removing the supernatant, the obtained POSS-Se was washed with 12 mL of water and 21 mL of CH_3_CN to remove residual desorbents. Then the recycled POSS-Se was utilized for the next adsorption experiment. The recycling experiments were performed 10 times.

## 3. Results and Discussion

### 3.1. Synthesis and Characterization

As illustrated in Scheme 1a, octa((4-benzyl imidazolium chloride)ethenyl)silsesquioxane (M-2) was first synthesized by the quaternization reaction of octa((4-benzylchloride)ethenyl) silsesquioxane (M-1) with 1-methylimidazole in chloroform at reflux temperature for 24 h. Then M-2 further reacted with excess selenium powder in the presence of K_2_CO_3_ at room temperature, affording the final product POSS-Se as a white solid in a high yield. In addition, a model compound (MC) was also prepared using a similar method from 4-vinylbenzyl chloride, 1-methylimidazole, and selenium powder [31] (Scheme 1b). Unlike the good solubility of the model compound in common solvents, POSS-Se is only soluble in several solvents, including chloroform, dichloromethane, dimethylsulfoxide, and *N*,*N*’-dimethyl formamide, but insoluble in methanol, diethyl ether, acetone, and water. This difference is obviously due to the presence of the inorganic–organic Si–O–Si cage and the bulky structure of POSS-Se.

**Figure 1:**
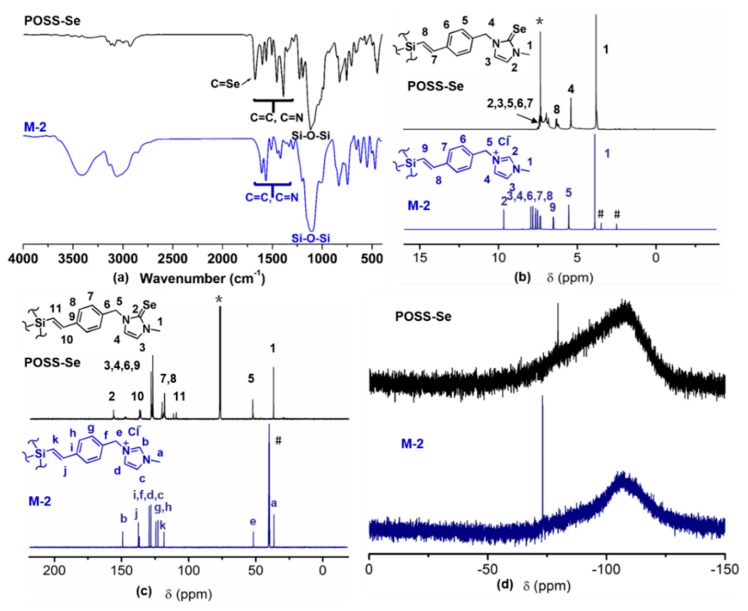
FT-IR spectroscopy (**a**), ^1^H NMR (**b**), ^13^C NMR (**c**), and ^29^Si NMR (**d**) of octa((4-benzyl imidazolium chloride)ethenyl)silsesquioxane (M-2) and POSS-Se. The asterisk and pound denote CDCl_3_ or *d*_6_-DMSO.

**Figure 2:**
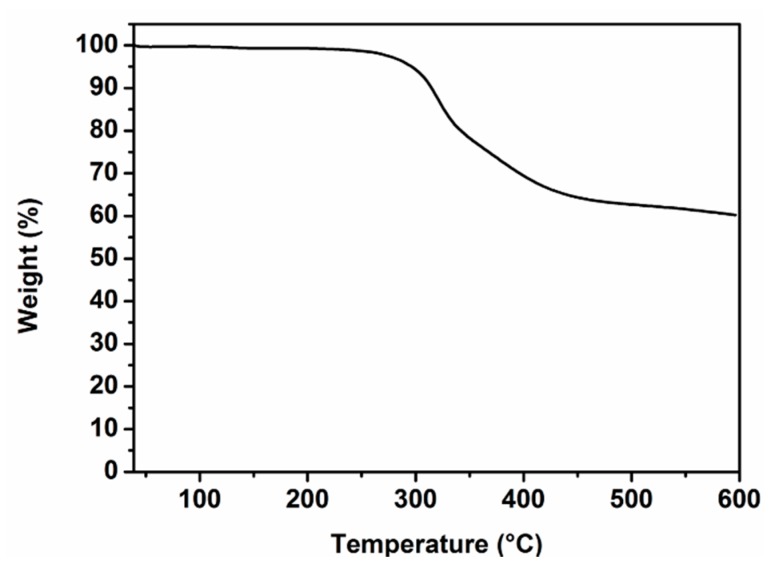
TGA curve of POSS-Se under N_2_ in the range of room temperature to 600 °C with a heating rate of 10 °C min^−1^.

The structures of POSS-Se and M-2 were fully determined by FT-IR, ^1^H NMR, ^13^C NMR, ^29^Si NMR, and elemental analysis. As shown in Figure 1a, the absorption bands from 1608 to 1450 cm^−1^ are associated with C=C or C=N stretching vibrations from vinyl, phenyl, and imidazolium groups in POSS-Se or M-2. The peak at 1678 cm^−1^ is derived from the C=Se stretching vibration in POSS-Se. The strong peaks at ca. 1100 cm^−1^ are obviously assigned to the characteristic Si–O–Si stretching vibration. In the ^1^H NMR spectra (Figure 1b), the signals, which are assigned to the methyl hydrogens, are observed at 3.89 and 3.72 ppm for M-2 and POSS-Se. The peaks at 5.54 and 5.35 ppm are attributed to the methylene hydrogens from M-2 and POSS-Se. The peak at 9.67 ppm is attributed to the imidazolium hydrogens (–N=C*H*–N–) in M-2 and disappears in POSS-Se, indicating the successful reaction between M-2 and selenium powder. In the ^13^C NMR spectra (Figure 1c), the signals derived from the methylene carbons are observed at 51.7 and 52.9 ppm for M-2 and POSS-Se. The peak at 156.6 ppm is ascribed to the imidazolium carbons (–N=*C*H–N–), while the formed selenone carbons gives rise to a peak at 156.6 ppm. The ^29^Si NMR spectra reveal one signal at −73.1 and −79.5 ppm for M-2 and POSS-Se (Figure 1d). Moreover, the found nitrogen content of POSS-Se fits well with the calculated one as evaluated by elemental analysis. These results demonstrate the successful achievement of M-2 and POSS-Se. Additionally, POSS-Se exhibits a good thermal stability with *T*_d, 5%_ (5% mass loss) of ca. 295 °C, which was evaluated by thermal gravimetric analysis (TGA) under N_2_ in the range of room temperature to 600 °C with a heating rate of 10 °C min^−1^ (Figure 2).

### 3.2. Detection of Hg^2+^ Ions by POSS-Se

As the selenium element is in the VIA group, which also contains the sulfur element, selenium resembles sulfur in chemical activity and physical properties. It is well-known that sulfur-based adsorbents have found important applications in the detection and/or adsorption of Hg^2+^ ions, which is one of the most toxic and hazardous heavy metal ions and greatly threatens environmental safety and human health [34,35]. However, there are few examples of selenium-based Hg^2+^ sensors or adsorbents [31,36,37]. Thus, POSS-Se was used to detect and adsorb Hg^2+^ ions in aqueous solutions.

**Figure 3:**
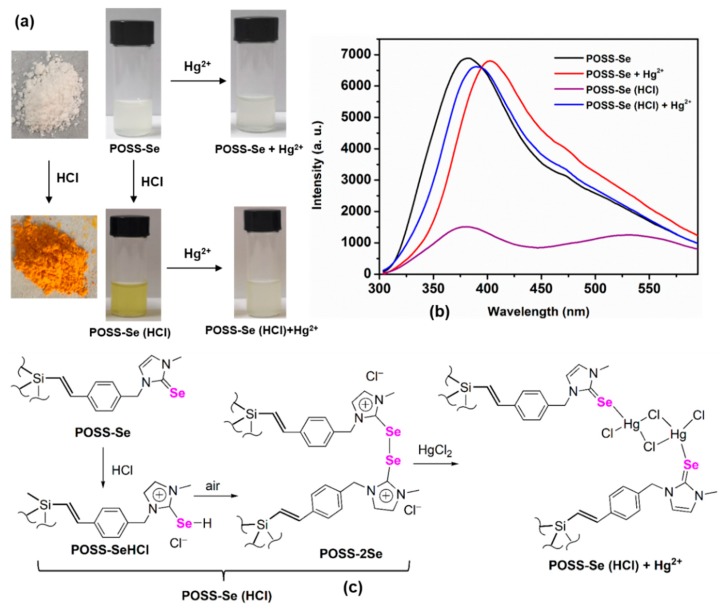
(**a**) The photographs of POSS-Se and the acid treatment product of POSS-Se (POSS-Se (HCl)) in the solid state, and their suspensions in water (0.1 mg mL^−1^) before and after the addition of Hg^2+^ ion (100 ppm); (**b**) The fluorescence emission spectra of POSS-Se, POSS-Se + Hg^2+^, POSS-Se (HCl) and POSS-Se (HCl) + Hg^2+^ suspension in water (*λ*_ex_ = 280 nm); (**c**) The possible mechanism of detecting Hg^2+^ by POSS-Se after treating with HCl.

Fortunately, POSS-Se can be well-dispersed in water, which is beneficial for the detection. It was found that there is no color change upon the addition of Hg^2+^ (100 ppm) into POSS-Se suspension in water (0.1 mg mL^−1^) (Figure 3a, upper right). Previously, Son et al. found that the acid treatment of a selenone compound resulted in the formation of a red-orange colored dimerized salt and this characteristic also applied to polymer articles, imparting them a detection ability towards Hg^2+^ by observing a vivid color change [31,36]. Motivated by this finding, POSS-Se was treated with 1.0 M hydrochloric acid solution (HCl) for 12 h, resulting in a red-orange solid (POSS-Se (HCl), Figure 3a left). After the acid treatment, the product become more hydrophilic due to the generated ionic character. Then POSS-Se (HCl) was dispersed in water and appeared as a dark yellow suspension at a low concentration of 0.1 mg mL^−1^. Upon the addition of Hg^2+^ ions (100 ppm), the suspension showed an obvious color change from dark yellow to pale yellow within 30 s (Figure 3a, low right). This color transition allows the “bare-eye” detection of Hg^2+^ by POSS-Se (HCl). In addition, upon the completion of the detection, the formed complex can be easily obtained by filtering the suspension, indicating its potential as a candidate for the removal of Hg^2+^ from aqueous solutions (see the next part).

To obtain a deep understanding of the detection process, the fluorescent emission spectra of POSS-Se suspensions before and after the detection were measured. As shown in Figure 3b, POSS-Se suspension in water exhibits a broad emission band with the maximum wavelength (*λ*_max_) at 385 nm. Upon the addition of Hg^2+^, no obvious change of emission intensity and only a small red-shift of ca. 20 nm were observed. This finding could explain why POSS-Se is not sensitive for the detection of Hg^2+^. In contrast, the acid treatment of POSS-Se leads to a large fluorescence quenching, indicating that POSS-Se has successfully reacted with HCl and thus a vivid color change occurred. Interestingly, the fluorescence quenching is recovered after adding Hg^2+^ and the resulting emission spectrum is nearly the same as that of POSS-Se + Hg^2+^. This finding means that Hg^2+^ ions have reacted with the acid product POSS-Se (HCl) and afford the same product, which was formed by the direct reaction of POSS-Se and Hg^2+^.

The detection mechanism was previously demonstrated in imidazoline-2-selone [38] and analogous selenone-modified submicro-polymer particles [31]. As illustrated in Figure 3c, the acid treatment of POSS-Se first generates ionic POSS-SeHCl, which is a selenol derivative and very sensitive to air and shows a facile spontaneous oxidation to a dimerization to form the Se–Se bond (POSS-2Se) at room temperature under air [38] (These two steps were named POSS-Se (HCl)). Then dark yellow POSS-2Se suspension in water reacts with HgCl_2_, resulting in the formation of Hg–Se coordination bonds accompanied by the destruction of Se–Se bonds. Thus the color turns pale yellow. In one word, the detection of Hg^2+^ by POSS-Se is due to the Se–Se formation by acid-treatment and subsequent coordination-induced cleavage of Se–Se bonds upon the addition of Hg^2+^. From the viewpoint of fluorescence variation, the fluorescence quenching after the acid treatment of POSS-Se can be explained by the formed ionic units, which may act as a fluorescence quencher. After adding Hg^2+^, the ionic units were converted to the initial state, i.e., selenone, while the Hg–Se coordination bonds were formed. Subsequently, the fluorescence intensity was recovered.

**Figure 4:**
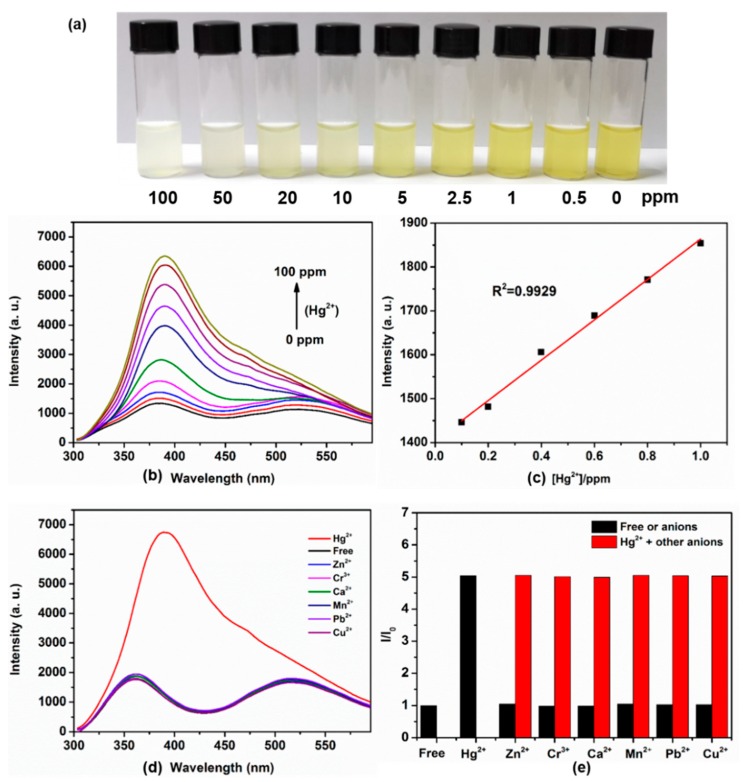
(**a**) The photographs of POSS-Se (HCl) suspension in water (0.1 mg mL^−1^) with various concentrations of Hg^2+^ ions (0–100 ppm); (**b**) The fluorescence emission spectra of POSS-Se (HCl) suspension with various concentrations of Hg^2+^ ions (*λ*_ex_ = 280 nm); (**c**) Linear relationship between emission intensity of POSS-Se (HCl) suspension in the presence of Hg^2+^ ions. σ = Standard deviation of blank measurement = 1.30; (**d**) The fluorescence emission spectra of POSS-Se (HCl) suspension with various metal ions (100 ppm, *λ*_ex_ = 280 nm); (**e**) Detection selectivity of POSS-Se (HCl) suspension (0.1 mg mL^−1^) towards Hg^2+^ ions in the presence of other metal ions in aqueous solutions. Black bars represent POSS-Se (HCl) suspension or POSS-Se (HCl) suspension with one metal ion. Red bars represent the subsequent of Hg^2+^ ions (100 ppm) to the suspensions.

To further investigate the detection behavior, the detection was performed by adding successive concentrations from 0 to 100 ppm of Hg^2+^ to POSS-Se (HCl) suspension in water. The colors of the suspensions gradually turned from dark yellow to pale yellow with increasing concentrations of Hg^2+^ (Figure 4a), while the fluorescence intensity gradually strengthened (Figure 4b). This feature allows this sensor to realize not only the “bare-eye” detection of Hg^2+^, but also a “turn-on” fluorescence detection. Moreover, the limit of detection was calculated from the slope using a linear relationship between the fluorescence intensity at 385 nm of POSS-Se (HCl) and the concentrations of Hg^2+^ ions. POSS-Se (HCl) exhibits a high sensitivity towards Hg^2+^ with a low LOD of 8.48 ppb (Figure 4c). The acquired value is comparable to or higher than many reported sensors [39], such as Fe_3_O_4_@TiO_2_–L (L=2,6-bis(2-thienyl)pyridine, 1.40 ppm) [40], thioether-based fluorescent covalent organic framework (COF-LZU8, 25.0 ppb) [41], metal-organic framework (NH_2_-MIL-53(Al), 30.13 ppb (0.15 μM)) [42], nature fibers modified by polymer chains (MA−SAPQA@NFs, 16.72 ppb (0.08 μM)) [43], silica modified by an anthracene excimer (SiO_2_@PBATPA, 37 ppb) [44], and vesicular chemical sensor (10.64 ppb (53.0 nM)) [45], but lower than some sensors such as rhodamine B selenolactone (4.62 ppb (53.0 nM)) [37], fluorescent phosphane selenide (0.18 ppb) [46], and sulfur-doped porous reduced graphene oxide [47] (Table 1). The lower performance may be due to the heterogenous detection by POSS-Se (HCl), which is insoluble in water, in contrast to the homogenous detection by most sensors, which are commonly soluble in water.

Another important factor of Hg^2+^ sensors is the selectivity among various metal ions. Thus, the detection behaviors of POSS-Se (HCl) towards common metal ions such as Zn^2+^, Cr^3+^, Ca^2+^, Mn^2+^, Pb^2+^ and Cu^2+^ were first performed in water with a concentration of 100 ppm. As shown in Figure 4d, no significant change of fluorescence emission was found. Then the competitive experiments were conducted in the presence of Hg^2+^ (100 ppm) mixed with various metal ions (Zn^2+^, Cr^3+^, Ca^2+^, Mn^2+^, Pb^2+^, and Cu^2+^, 100 ppm). It was found that the fluorescence enhancement changes are negligible after adding other metal ions (Figure 4e), thereby indicating its good selectivity and suggesting that POSS-Se after the acid-treatment could serve as a promising sensor for selectively detecting Hg^2+^.

### 3.3. Adsorption of Hg^2+^ Ions by POSS-Se

Developing an efficient adsorbent for simultaneous detection and removal of Hg^2+^ is highly desirable. The excellent sensing performance of POSS-Se towards Hg^2+^ inspired us to examine its ability to capture Hg^2+^ from aqueous solutions. As mentioned above, it is necessary to acid-treat POSS-Se for the detection of Hg^2+^ because there is no obvious color or fluorescence change in the sensing Hg^2+^ process by POSS-Se (Figure 3b). However, the small red-shift found in the fluorescence emission spectra of POSS-Se in the presence of Hg^2+^ suggests that the interaction between POSS-Se and Hg^2+^ also occurred, indicating its possibility for the adsorption of Hg^2+^. Therefore, a comparison experiment was performed by adsorbing Hg^2+^ ions (1000 ppm) using POSS-Se and the acid product POSS-Se (HCl) as the adsorbents as suspension in water (1 mg mL^−1^). POSS-Se and POSS-Se (HCl) were determined to be 639 and 539 mg g^−1^, respectively. The lower capacity after the acid treatment is due to the increased molecular weight, while the capacity is calculated in terms of “mg g^−1^”. This finding reveals that for the adsorption of Hg^2+^, the acid treatment of POSS-Se is not necessary. Thus we used POSS-Se as the adsorbent to investigate the adsorption behavior towards Hg^2+^. 

**Figure 5:**
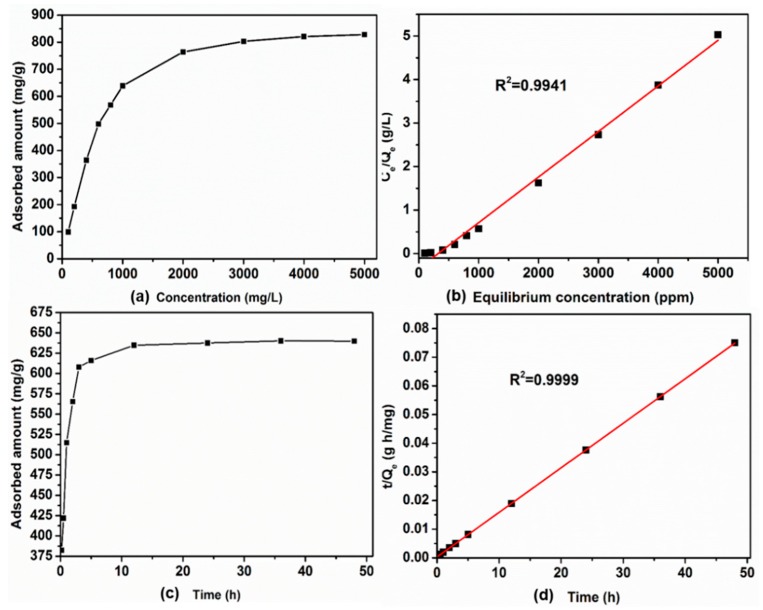
(**a**) Hg^2+^ isotherm for POSS-Se suspension in water (1 mg mL^−1^); (**b**) The Langmuir isotherm model for the adsorption of Hg^2+^; (**c**) Adsorption curve of Hg^2+^ in aqueous solutions (1000 ppm) versus contact time by POSS-Se suspension in water (1 mg mL^−1^); (**d**) The pseudo-second-order kinetic model plots for the adsorption.

To evaluate the overall capacity of POSS-Se for Hg^2+^, equilibrium values were collected by adding Hg^2+^ aqueous solutions with the initial concentrations from 50–5000 ppm. As shown in Figure 5a, the adsorption capacity increases with an increment of initial Hg^2+^ concentrations, which can be explained by the driving force of the concentration gradient. The equilibrium adsorption isotherm data was well-fitted with the Langmuir model, yielding a correlation coefficient of 0.9941 (Figure 5b). Moreover, POSS-Se was determined to have a capacity of 952 mg g^−1^ of Hg^2+^ (the capacity of POSS-Se (HCl) is 907 mg g^−1^, which was determined under the same method to that of POSS-Se). This value is higher than most reported Hg^2+^ adsorbents, such as NH_2_-MIL-53(Al) (153.85 mg g^−1^) [42], MA−SAPQA@NFs (218.21 mg g^−1^) [43], sulfur-doped porous reduced graphene oxide (829.27 mg g^−1^) [47], SiO_2_-G0-SA∼SiO_2_-G2.0-SA (363.07 mg g^−1^) [51], and TAPB-BMTTPA-COF (734 mg g^−1^) [53], although some of them have lower LODs compared to that of POSS-Se (HCl) (Table 1). 

The adsorption capacities with respect to contact time were also studied with the initial Hg^2+^ concentration of 1000 ppm. It can be observed that the initial Hg^2+^ adsorption rate was rapid during the early stage of the adsorption within 3 h, and the capacity reached 608 mg g^−1^. After that, the adsorption rate gradually slowed down, leading to equilibration at ~24 h, while the capacity reached 640 mg g^−1^. The initial high rate may be caused by the higher Hg^2+^ concentration gradient and ready accessibility of Hg^2+^ to the binding sites. It could be speculated that the dispersibility of POSS-Se in water would become worse with an increment of the adsorption amount of Hg^2+^. Moreover, the adsorption kinetic process was well fitted with the pseudo-second-order kinetic model, yielding a correlation coefficient of 0.9999 (Figure 5d).

**Figure 6:**
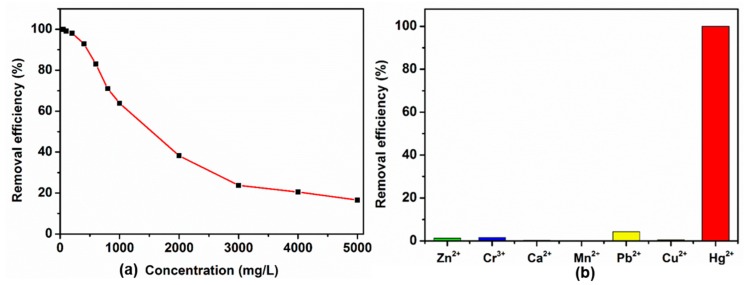
(**a**) The removal efficiency of Hg^2+^ with concentration in the range of 50–5000 ppm by POSS-Se suspension in water (1 mg mL^−1^); (**b**) The selective removal efficiency of the co-existence of Hg^2+^ (100 ppm) and other metal ions (100 ppm) by POSS-Se suspension in water (1 mg mL^−1^).

In addition to the high Hg^2+^ uptake, the removal efficiency is also important for Hg^2+^ adsorption. The efficiency was estimated by using POSS-Se (1 mg mL^−1^) to remove Hg^2+^ ions with a concentration in the range of 50–5000 ppm. As expected, on increasing the concentration of Hg^2+^, the removal efficiency gradually decreases (Figure 6a). However, at a low concentration, the efficiency is very high. For example, the original Hg^2+^ level at 50 and 10 ppm can be reduced to 3 ppb and 0.5 ppb (well below the permissible level (2 ppb) for drinkable water) with the efficiency of 99.994% and 99.995% within 12 h. In addition, the effect of the co-existence of various metal ions, including Zn^2+^, Cr^3+^, Ca^2+^, Mn^2+^, Pb^2+^, and Cu^2+^ on the removal efficiency of Hg^2+^ was also studied. It was found that the presence of other metal ions barely affected the removal efficiency of Hg^2+^ by POSS-Se (Figure 6b).

**Figure 7:**
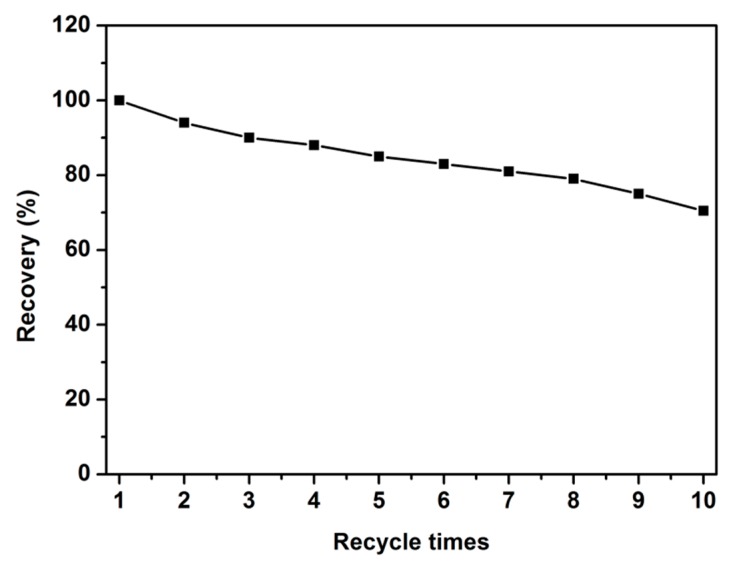
Plot of recovery ratio of POSS-Se suspension as a function of recycle times.

Recyclability of adsorbents is important for industrial applications, especially in terms of cost. Therefore, the recyclability of POSS-Se was investigated in the adsorption of Hg^2+^ ions. By using sodium diethyldithiocarbamate (DDTC-Na) as the chelating reagent [58], the Hg^2+^ ions can be easily removed from POSS-Se within 30 min. Thus, the adsorption–desorption process was repeated 10 times by using 5 mL of suspension in water containing Hg^2+^ ions (1000 ppm) and POSS-Se (1 mg mL^−1^) for 30 min as the standard experiment. The relative adsorption capacity was calculated on the basis of the adsorption capacity determined in the first cycle as a benchmark before and after adsorption. In the first cycle, the adsorption capacity was regarded as 100%. After desorbing the Hg^2+^ ions by DDTC-Na, the recovery of the relative adsorption capacity was 94%. After ten cycles, the capacity recovery was 70.5% (Figure 7). We speculate that the decrease in recovery can be attributed to the weight loss of used POSS-Se samples during washing and centrifugation process. These results reveal that POSS-Se is an efficient adsorbent for the removal of Hg^2+^ ions from the aqueous solutions and can also be recycled. 

## 4. Conclusions

In summary, a novel selenone-functionalized polyhedral oligomeric silsesquioxane (POSS-Se) was prepared by the reaction of an imidazolium-containing POSS with selenium powder. After an acid treatment of POSS-Se, the product POSS-Se (HCl) suspension in water can serve as an efficient sensor for the detection of Hg^2+^ with two models, including “bare-eye” visualization of the color change from dark yellow to pale yellow, or by observing the “turn-on” fluorescence phenomenon. This finding is due to the Se–Se formation by acid-treatment and subsequent coordination-induced cleavage upon the addition of Hg^2+^ ions. This sensor shows a high sensitivity with a low limit of detection (8.48 ppb), which is comparable to many reported Hg^2+^ sensors. Moreover, POSS-Se possesses a high adsorption capacity (952 mg g^−1^) for Hg^2+^ ions in aqueous solutions without the requirement of acid treatment. The performance is better than most reported adsorbents for Hg^2+^. These results reveal that this novel selenone-functionalized POSS can be potentially utilized as a promising candidate for the detection and removal of Hg^2+^ from aqueous solutions. Further investigation will focus on the synthesis of novel selenium-containing POSS-based materials and exploration of their applications.

## Figures and Tables

**Table 1:** The limit of detections (LODs) and capacity comparison of POSS-Se with other adsorbents.

Samples	LOD/ppb	Adsorption Capacity/mg g^−1^	Ref.
POSS-Se	8.48 *^a^*	952	This work
Fe_3_O_4_@TiO_2_–L	1.40 ppm	15 *^b^*	[40]
Thiol-based fluorescent covalent organic framework (COF-LZU8)	25.0	-	[41]
SiO_2_@PBATPA	37	482	[44]
NH_2_-MIL-53(Al)	30.13 *^b^*	153.85	[42]
Salicylaldehyde-containing polymeric quaternary ammonium chains onto the natural fibers (MA−SAPQA@NFs)	16.72 *^b^*	218.21	[43]
Vesicular chemical sensor	10.64 *^b^*		[45]
PDI–BODIPY dendrimer	4.02 *^b^*	-	[48]
Mesoporous conjugated adsorbent	3.69	172.61*^b^*	[49]
Rhodamine B selenolactone	4.62	-	[37]
fluorescent phosphane selenide	0.18	-	[46]
Sulfur-doped porous reduced graphene oxide	0.1	829.27	[47]
Histidine conjugated perylene diimide bolaamphiphile	0.01 ppq	-	[50]
SiO_2_-G0-SA∼SiO_2_-G2.0-SA	-	363.07 *^b^*	[51]
Polymer particles containing imidazoline selenones	-	110	[31]
Cellulose and thiol-based adsorbent	-	404.95	[52]
TAPB-BMTTPA-COF	-	734	[53]
Chelating fiber ACHF	-	785.2	[54]
Thiol-functionalized [Cu_3_(BTC)_2_]_n_	-	714.29	[55]
COF-S-SH	-	1350	[56]
Polydopamine nanospheres	-	1861.72	[57]

^a^ The LOD is calculated based on the detection behavior of POSS-Se after acid treatment. ^b^ The capacity was re-calculated based on the values in literatures.
